# How to Achieve High Encapsulation Efficiencies for Macromolecular and Sensitive APIs in Liposomes

**DOI:** 10.3390/pharmaceutics13050691

**Published:** 2021-05-11

**Authors:** Kirsten Ullmann, Gero Leneweit, Hermann Nirschl

**Affiliations:** 1 Institute of Mechanical Process Engineering and Mechanics, Process Machines, Karlsruhe Institute of Technology (KIT), 76131 Karlsruhe, Germany; hermann.nirschl@kit.edu; 2 Carl Gustav Carus-Institute, Association for the Promotion of Cancer Therapy, 75223 Niefern-Oeschelbronn, Germany; gero.leneweit@carus-institut.de; 3 Abnoba GmbH, 75223 Niefern-Oeschelbronn, Germany

**Keywords:** encapsulation efficiency, liposomes, phospholipids, fluorocarbon, nano-emulsions, active pharmaceutical ingredients

## Abstract

This research highlights the capacity of a newly introduced centrifugation process to form liposomes from water-in-fluorocarbon nano-emulsions stabilized with phospholipids to incorporate macromolecular and sensitive active pharmaceutical ingredients (API). The encapsulation efficiency of the produced liposomes, incorporating fluorescein-sodium, bovine serum albumin and fluorecein isothiocyanate dextran as model APIs, is determined by applying Vivaspin^®^ centrifugation filtration and quantified by UV-Vis spectroscopy. It was found that higher densities of the fluorocarbons used as the hydrophobic phase enable a higher encapsulation efficiency and that an efficiency of up to 98% is possible depending on the used phospholipid. Among the engineering aspects of the process, a comparison between different membrane substances was performed. Efficiency increases with a higher phospholipid concentration but decreases with the addition of cholesterol. Due to the higher bending modulus, liposome formation is slowed down by cholesterol during liposome closure leading to a greater leakage of the model API. The encapsulation of bovine serum albumin and dextran, both investigated under different osmotic conditions, shows that an efflux negatively affects the encapsulation efficiency while an influx increases the stability. Overall, the process shows the potential for a very high encapsulation efficiency for macromolecules and future pharmaceutical applications.

## 1. Introduction

To this day, cancer remains one of the most severe diseases, even though mortality has decreased overall due to steady reductions in smoking and advances in early detection and treatments for patients [1]. However, some death rates increased from 2012–2016, such as for cancers of the liver, pancreas, uterus, brain and nervous system [1]. Common methods in cancer therapy, such as the use of chemotherapeutic substances, are not specific to cancer cells but affect healthy cells as well and harm the patient to a large extent. A way to prevent the unnecessary harm of the human body is the specific targeting of the tumour by using, for example, fusion protein engineered antibodies [2]. These approaches still face the problem of degradation because the endogenous defence detects the antibodies that carry the drug as an intruder. A different approach is the encapsulation of pharmaceutical ingredients in liposomes which can be delivered to the target cell without previous degradation [3]. Liposomes consist of phospholipids that play a key role in the food and pharma industry because of their ubiquity in all organisms and their absolute safety [4]. They are often utilized as natural emulsifiers. Using PLs as emulsifiers or building blocks, they contribute to the properties of the delivery system. The amphiphilic characteristics of the phospholipids are the constitutive base of all biological membranes and allow the carrying of an active ingredient inside, while transport through the blood circulation is possible without provoking an immediate immune response [4].

Manufacturing methods for liposomes and other drug delivery vehicles have been the subject of intensive research for over 30 years. A conventional laboratory method for the production of liposomes is the film method, in which the phospholipids, possibly with a hydrophobic active ingredient, are dissolved in a solvent, e.g., ethanol or a mixture of methanol and chloroform. The solvent is then removed in the rotary evaporator so that the lipids form ordered bilayers at the wall of the flask. The hydrophilic active ingredient is encapsulated by adding the aqueous solvent. The lipids start swelling and form a heterogeneous suspension of multilamellar vesicles (liposomes) in which the active ingredient is encapsulated. Depending on the solubility of the active ingredient, the continuous phase can be aqueous or hydrophobic [5,6]. 

The production of uni-lamellar liposomes with defined sizes can be achieved in different ways. One possibility is the extrusion process, in which the solution is forced through nano-meter sized pores in a membrane under high pressure. The multilayers are fractured as they enter the nanopores and multilamellar vesicles are converted into uni-lamellar liposomes [7]. The membrane pore size controls the size of the resulting liposomes [8]. Another alternative for large production volumes is the use of a homogenizer [9]. The liposome suspension passes through the homogenizer several times and the liposomes decrease in size with each pass. The minimally achievable sizes are 20 nm [10,11]. 

Based on their mechanism of action all the above methods are reliable regarding size mono-dispersity, but the concentration of active ingredients inside is the same as outside at the moment of vesicle closure. The encapsulation efficiency found in the literature for high molecular weight molecules is only in the low double-digit range and varies from about 2–50% in cases of affinity between active ingredients and liposomal membrane [12,13]. Higher efficiencies have not yet been established as so-called ‘remote loading’ is only feasible for small molecules that diffuse through phospholipid bilayers, but not for macromolecular APIs. A “high” encapsulation efficiency is proposed by Xu et al. who use freeze-thaw cycles for entrapment of proteins [14]. Yet the proposed method does not exceed the 50% level either. Furthermore, the use of solvents is necessary for the majority of the manufacturing methods mentioned. However, even small traces of solvents are undesirable for application as a carrier for active ingredients in the pharmaceutical or food sector as they destabilize and degrade many active ingredients, especially peptides or proteins. A comprehensive removal of possible solvent residues in the production process is therefore extremely costly and time-consuming as is the removal of the API which is not encapsulated and thus, found freely in the solution, needing additional separation and purification steps [15]. 

Engineering methods, however, are not common even though they have a great potential for large scale application. Pautot et al. were the first to introduce a centrifugation method where the droplets of a water-in-oil (*w*/*o*) nano-emulsion transfer to a second aqueous phase to create a bilayer [16]. While this method works for large uni-lamellar vesicles of between 1 and 10 µm, several limitations exist such as an appropriate size for pharmaceutical applications, the stability of nano-emulsions, and oranogel formation between the three phases (water, oil and phospholipids). A smaller size of liposome was achieved by de Matos et al. [17]. However, the phase transfer and encapsulation efficiencies were still insufficient. Previous research tackled the above problems and revealed that several advantages regarding the centrifugation process were achieved by using a fluorocarbon as hydrophobic phase instead of a hydrocarbon (such as squalene or dodecane, used previously); hindrance by interfacial tension during phase transfer is compensated for by the much stronger density difference between the hydrophobic and aqueous phase (Δρ ≈1 g/cm³; ρ_water_ = 0.998 g/cm³; ρ_fluorocarbon_ = 2.03 g/cm³), enabling transfer. Organogel formation is no longer detectable. Nano-emulsion droplets are stable for several weeks and liposome production is successful [18]. Because of the heavier hydrophobic phase, the aqueous droplets float up instead of sedimenting during centrifugation, which enables easy removal of the liposomal suspension from the top.

The question remains as to whether a high encapsulation efficiency is possible by applying the centrifugation process. While De Matos et al. used the centrifugation method to produce asymmetric liposomes and encapsulated nucleic acids, they used squalene for the preparation of nano-emulsions leading to a plasmid encapsulation of 10–15% [17]. Hence, an evaluation of the encapsulation efficiency of the formed liposomes from the water-in-fluorocarbon (w/fc) nano-emulsion remains to be carried out.

The aim of this work is to show the capacity of the process for high encapsulation efficiency by using different fluorocarbons and the phospholipids DPPC, DPPG and DMPC, evaluated with fluorescein-sodium (FS) as a low molecular weight hydrophilic marker, as well as bovine serum albumin (BSA) and fluorescently labelled dextran as high molecular weight protein and polymer, respectively. The sequence of different analytical detection methods was evaluated for its capacity to determine the amount of the encapsulated model API, while being able to trace not only the API surrogates itself but also other compounds such as phospholipids and the fluorocarbon phase.

## 2. Materials and Methods

### 2.1. Materials

Phospholipids utilized for all experiments were provided by Lipoid (Ludwigshafen, Germany). The synthetic phospholipids 1,2-dimyristoyl-*sn*-glycero-3-phostphatidylcholine (DMPC), 1,2-dipalmitoyl-*sn*-glycero-3-phosphatidylcholine (DPPC) and 1,2-dipalmitoyl-*sn*-glycero-3-phospho-rac-glycerol (DPPG) were received in powder form. Cholesterol (chol.) was purchased from Carl Roth (Karlsruhe, Germany). Perfluoro-perhydro-phenanthrene (C_14_F_24_) was purchased from F2 Chemicals (Preston, UK) and has a density of 2.03 g/cm³ and a refractive index (RI) of 1.331. For comparison, the fluorocarbons perfluoro-heptane (C_7_F_16_, RI = 1.26, ρ = 1.72 g/cm³), perfluoro-1,3-dimethylcyclohexane (C_8_F_16_, RI = 1.2895, ρ = 1.83 g/cm³) and perfluoro-methyl-decalin (C_11_F_20_, RI = 1.3195, ρ = 1.92 g/cm³) from F2 Chemicals were also tested. Phosphate buffer consisted of a 1:4.2 mixture of sodium di-hydrogen phosphate (NaH_2_PO_4_) and di-sodium hydrogen phosphate (Na_2_HPO_4_, both Carl Roth, Karlsruhe, Germany). Encapsulation efficiency was measured via fluorescein-sodium (FS, Carl Roth, Karlsruhe, Germany), BSA (VWR International GmbH, Darmstadt, Deutschland) and fluorescein isothiocyanate-dextran (FITC-D, M_w_ = 70 kDa, Merck, Darmstadt, Germany). For phospholipid quantification, perchloric acid (70%) was purchased from Carl Roth (Karlsruhe, Germany). Further chemicals used for the phosphorus assay were ascorbic acid, hexa-ammonium molybdate and sodium dihydrogen phosphate as a standard phosphate solution (all Carl Roth, Karlsruhe, Germany).

### 2.2. Preparation of Aqueous Lipid Stock Suspension

Lipid suspensions were prepared with different concentrations of phospholipids in 1 mL with 15 mM phosphate buffer if not stated otherwise. The preparation was performed in micro reaction tubes (Eppendorf, Hamburg, Germany). Ultrasound provided by a 3 mm sonotrode tip (Digital Sonifier 450, Branson Ultrasonic, Danbury, CT, USA) was used for the dispersion of phospholipids with a 100% cycle and an amplitude of 10% for 10 s followed by a 50% cycle and 10% amplitude for 10 min. The temperature was kept constant at 30 °C. To allow the determination of the encapsulation efficiency, fluorescein-sodium was added to the stock suspension at a concentration of 10 g/L, BSA at a concentration of 100 g/L, and FITC-D in a concentration of 50 g/L. For mixtures of phospholipids and cholesterol the molar ratio amounted to 60:40. These aqueous media containing dissolved model active ingredients and dispersed lipids were used as the aqueous phase for the generation of w/fc nano-emulsions as the next step of liposome production.

### 2.3. Preparation of w/fc Nanoemulsions and Liposomes

The w/fc nano-emulsion contained 1% dispersed phase (aqueous lipid stock suspension, see above) and was used to produce liposomes via centrifugation. The hydrophobic phase consisted of the fluorocarbon perfluoro-perhydro-phenanthrene (C_14_F_24_), if not stated otherwise. The droplet size averaged around 180 nm and was found to be suitable for liposome production [18]. 

**Figure 1:**
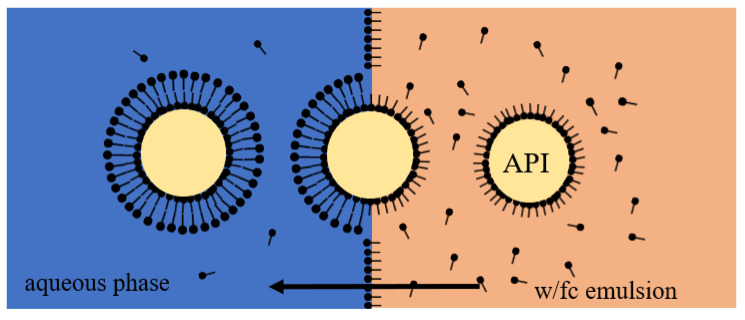
Schematic illustration of the centrifugation step to produce liposomes. Water droplets of a w/fc nano-emulsion containing the model API ascend towards a second aqueous phase via centrifugation. During the transfer, a second outer lipid leaflet forms the liposome by covering the transferred emulsion droplet. Adapted from [18], Published by Advanced Materials Interfaces, 2020.

Liposomes were prepared by transferring the water droplets of the w/fc nano-emulsion containing the model API to a second aqueous phase via centrifugation (Figure 1).

**Figure 2:**
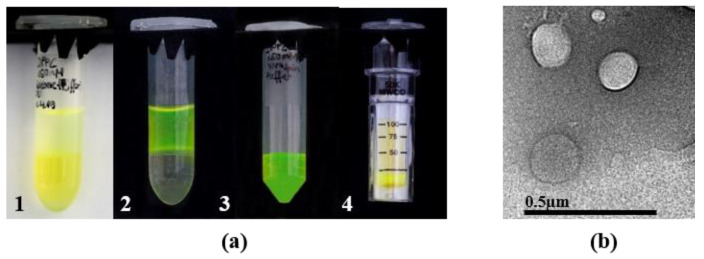
(**a**) Photographs of the centrifugation process performed in micro-reaction tubes. 1. w/fc emulsion containing FS with phosphate buffer as the aqueous phase on top. The yellow color on top is due to the reflection of the lower phase. 2. Complete transfer of the droplets to the upper aqueous phase. 3. Filtrate after centrifugation with the Vivaspin^®^. 4. Retentate (liposomes) left on the membrane of the Vivaspin^®^. (**b**) TEM picture of DPPC-liposomes (150 mM). Negative staining of the aqueous phase surrounding the liposomes was performed by ammonium molybdate.

Thus, the lipid-coated aqueous cores of the nano-emulsion became surrounded by outer lipid leaflets, incorporating the API. The centrifugation step was performed at 0 °C and 4000× *g* for 30 min (Eppendorf Centrifuge 5430 R, Eppendorf, Hamburg, Germany). Because of the higher density of the surrounding fluorocarbon, the lighter water droplets ascend to the upper aqueous phase. A photograph of the transfer including FS as a hydrophilic marker is depicted in Figure 2a. The produced liposomes show an average size of 60 nm, based on dynamic light scattering and small angle x-ray scattering measurements. A transmission electron microscopy (TEM) picture of DPPC-liposomes (150 mM) evidences the production of vesicles (Figure 2b, TEM CM12, co. Philips, The Netherlands). Zeta potential of liposomes was measured at a constant voltage of 50 mV with the Zetasizer Nano ZS (Malvern Instruments, Worcestershire, UK). For a detailed description of nano-emulsion and liposome preparation, as well as results of droplet and liposome size, please refer to Ullmann et al. [18].

### 2.4. Detection Method

For the detection of the model APIs FS, BSA and FITC-D inside the droplets, UV-Vis spectroscopy (DH-2000 Ocean Optics, Largo, FL, USA and UV-1900 Shimadzu, Kyoto, Japan) was chosen. The spectra of the initial phases—the fluorocarbon, the buffer, the emulsion, the stock solution and the diluted solution of either FS, BSA or FITC-D—were recorded beforehand. The encapsulated model API FS shows a significant absorption peak at 491 nm, BSA at 280 nm and FITC-D at 493 nm. The absorption spectra of different substances applied during the centrifugation process reveal that neither the fluorocarbon, the phosphate buffer, nor different DPPC concentrations of stock solutions expose a significant peak at the same position. PLs absorb at a wavelength of 230 nm. 

**Figure 3:**
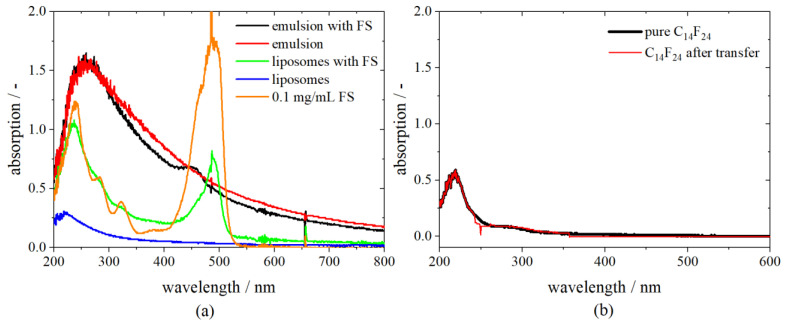
Absorption spectra of different compounds needed for the preparation of liposomes: (**a**) an emulsion with and without FS, liposomes prepared with and without FS and a pure stock solution of 0.1 mg/mL FS; (**b**) the spectrum of the pure fluorocarbon C_14_F_24_ and after the transfer of emulsion droplets.

As an example, the spectra of different compounds needed for the preparation of liposomes are shown in Figure 3a. A comparison between the absorption spectrum of an emulsion with FS and an emulsion without FS reveals that the fluorocarbon has a shielding effect on the dye. However, detection, as well as distinction, is possible as there is a small peak visible at 458 nm for the emulsion, including FS. 

After centrifuging the w/fc nano-emulsion and transferring of droplets to the upper aqueous phase, the lower hydrophobic phase (C_14_F_24_) was measured again. The fluorocarbon shows the same absorbance after centrifugation as in its pure condition, indicating that no FS nor phospholipids remain in the hydrophobic phase and the transfer is completed entirely (Figure 3b).

### 2.5. Encapsulation Efficiency 

To determine the encapsulation efficiency of the produced liposomes, FS was added at a concentration of 10 g/L to the lipid stock suspension, BSA at a concentration of 100 g/L and FITC-D at a concentration of 50 g/L. The final concentration of the model APIs in the nano-emulsion was 1:100 of the initial concentration as the emulsions were prepared with 1% (*v*/*v*) of the dispersed phase. 

After producing the nano-emulsion and centrifuging the droplets through the interface, the upper aqueous phase containing the liposomes and the model API is collected, and non-encapsulated FS is separated by Vivaspin 500^®^ (Sartorius, Göttingen, Germany). The Vivaspin 500^®^ has a capacity of 500 µL and a separation limit of 50 kDa. For BSA and FITC-D, a Vivaspin 2^®^ with a 100 kDa cut off was chosen. To separate liposomes from free FS, BSA, or FITC-D, the samples were centrifuged for 3 h with 4000× *g* at 0 °C. Liposomes remain in the filter module (retentate) while the non-encapsulated model API passes through the membrane and is quantified via the UV-Vis spectrometer (filtrate). 

The encapsulation efficiency considering the initial concentration $c_{0}$ is called ${\mathrm{EE}}_{\mathrm{theo}}$, and its value is provided in % and is determined by

(1) $${\mathrm{EE}}_{\mathrm{theo}}\;\left(\%\right)=\left(1-\frac{c_{F}}{c_{0}}\right)\cdot 100\;$$

where $c_{F}$ is the amount of free model API in the filtrate and $c_{0}$ is the initial concentration of API in the emulsion.

### 2.6. Recovery Rate (RR)

In addition to the measurement of the free model API after the separation step, the amount of the marker in the complete aqueous phase was detected, e.g., shown here for FS (RR_FS_). Perchloric acid destroys the liposomes and allows the measurement of the total amount of FS which should add up to 100% of the initial amount. To determine the total amount of FS compared to the initial amount, the liposome suspension was treated with 70% perchloric acid at a volume ratio of 1:1 for solubilisation of liposomes and quantified via UV-Vis spectroscopy at 435 nm according to a calibration curve with perchloric acid, respectively. Treatment took place for 10 min and 50 °C on a heated shaker. In addition, possible losses of FS at the wall of the reaction tube were taken into account by washing the tubes with perchloric acid and quantification by UV-Vis spectroscopy. The retentate (liposome remains on the Vivaspin membrane) was treated likewise with perchloric acid to specify the amount of FS. The quantification is summed up as follows and was performed for FS, dextran and phospholipids:

(2) $${\mathrm{RR}}_{x}\;\left(\%\right)=\left(\frac{c_{x,\mathrm{total}}}{c_{0}}\right)\cdot 100\;$$

where the index x stands for either FS, D (dextran) or PL (phospholipid), *c_x,total_* is the total amount of model API or phospholipids found in the upper aqueous phase after transfer, and c_0_ is the initial amount from the stock suspension. When referring to RR as a concentration, e.g., the RR of FS, it is defined as c_RR_ in mM.

### 2.7. Encapsulation under Different Osmotic Conditions

The efficiency of the encapsulation was monitored under different osmotic conditions inside and outside the liposomes. For this purpose, the phosphate buffer concentration was changed from 15 mM to a range between 83 mM and 250 mM, and no salt ions (0 mM).

### 2.8. Determination of Phospholipids

The concentration of phospholipids in the filtrate as well as possible losses and the concentration of phospholipids that are completely transferred were determined by phosphorus assay according to Fiske [19]. Perchloric acid (70%) destroys the phospholipid molecules while ascorbic acid and ammonium molybdate lead to a change of blue color under the presence of phosphorus.

## 3. Results and Discussion

### 3.1. Loss of Phospholipids

The losses of phospholipids during the process were monitored for the lipid DPPC at a concentration of 150 and 300 mM. It was found that a loss appears during sonication and that the stock suspension does not fully retain the initial concentration (cf. Table 1. (A) and (B)). Most phospholipids are found in the upper aqueous phase after centrifugation (cf. column (C)), as expected. In addition, small amounts remain on the wall of the reaction tube after the hydrophobic phase is removed (cf. column (D)). In that case the reaction tube was washed with the same volume of perchloric acid as the initial emulsion. In total, a loss of 30% is common for all phospholipids (cf. column (F)), which is mainly due to the greater amount of phospholipids that remain at the interface and cannot be completely collected.

### 3.2. Recovery Rate

For comparison, a stock suspension with FS was treated in the same way as a liposome suspension. Both absorption spectra show the same results after being heated up with perchloric acid, proving the completeness of FS release from liposomes and the consistency of the method. DPPG shows a bigger loss of FS during the liposome production process (RR_FS_ of 81%) while the RR_FS_ for liposomes produced with DPPC (with both 150 and 300 mM) is at 91% (Table 2). However, a concentration of 50 mM DPPC in the stock suspension only leads to a RR_FS_ of 48%. A concentration of only 50 mM was found to be too low for an adequate stabilization of w/fc nano-emulsions (cf. Ullmann et al. [18]). The phospholipid DMPC shows a similar low RR_FS_ of 45%, even at 150 mM. Nonetheless, taken into account that losses during sonication and transfer with a high surface to volume ratio are to be considered, an RR_FS_ of 91% is an unexpected high yield. 

For the depiction of data regarding the EE, the theoretical value E_theo_ was replaced with a corrected efficiency EE_cor_ based on the FS-concentration c_RR_ (or other model APIs, respectively) found after the transfer:

(3) $$E_{cor}\;\left(\%\right)=\left(1-\frac{c_{F}}{c_{\mathrm{RR}}}\right)\cdot 100\;$$

### 3.3. Effect of Different Fluorocarbons on Encapsulation Efficiency

**Figure 4:**
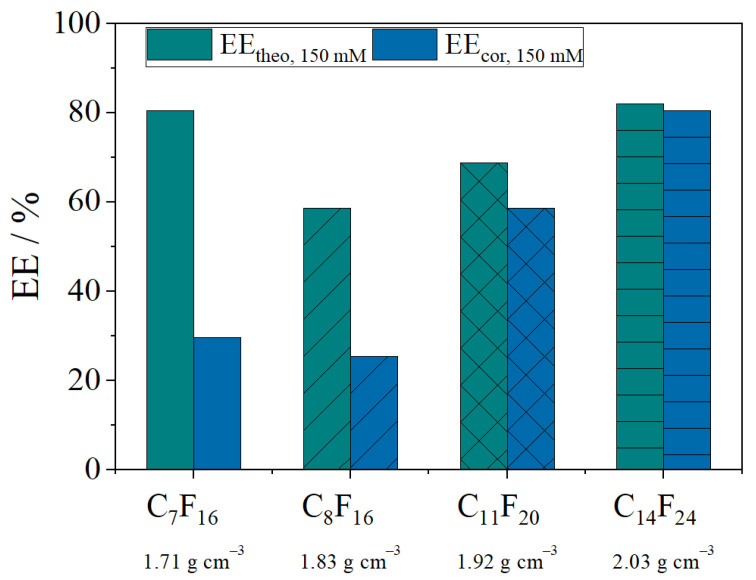
Effect of different fluorocarbons (perfluoro-heptane (C_7_F_16_), perfluoro-1,3-dimethylcyclohexane (C_8_F_16_), perfluoro-methyl-decalin (C_11_F_20_) and perfluoro-perhydro-phenanthrene (C_14_F_24_)) on the encapsulation of FS with a lipid stock suspension of 150 mM DPPC. The green bars show the EE_theo_ based on the initial amount of FS added to the stock suspension (Equation (1)), while blue bars depict the EE_cor_ (Equation (3)), based on the RR (Equation (2)). In addition, the densities of each fluorocarbon are listed.

Different fluorocarbons were examined regarding their contribution to encapsulate the model API FS, namely perfluoro-heptane (C_7_F_16_), perfluoro-1,3-dimethylcyclohexane (C_8_F_16_) and perfluoro-methyl-decalin (C_11_F_20_) and perfluoro-perhydro-phenanthrene (C_14_F_24_) (Figure 4). For a better comparison and classification, two different calculation methods were considered. EE_theo_ as in Equation (1) is based on the initial amount of FS while EE_cor_ (Equation (3)) uses the values of the RR for calculation. The latter takes losses of the API into account. Hence, a large difference between EE_theo_ and EE_cor_ demonstrates a poor encapsulation. The results reveal a significant distinction between EE_theo_ and EE_cor_ for the fluorocarbons C_7_F_16_ and C_8_F_16_. While for C_11_F_20_ the losses of FS are smaller, visual observations during the experiment showed an unstable nano-emulsion with droplets quickly moving to the upper phase without centrifugation. Figure 4 leads to the conclusion that the lower the density of the fluorocarbon phase the lower the encapsulation efficiency because losses are higher. The instability of nano-emulsions prepared with lighter fluorocarbons according to their molecular weight has been reported previously [20]. Thus, a higher loss of API is likely if the stability of nano-emulsions is already poor. As C_14_F_24_ shows the best results, it was applied for further experiments.

### 3.4. Influence of Different DPPC Concentrations and the Addition of Cholesterol

**Figure 5:**
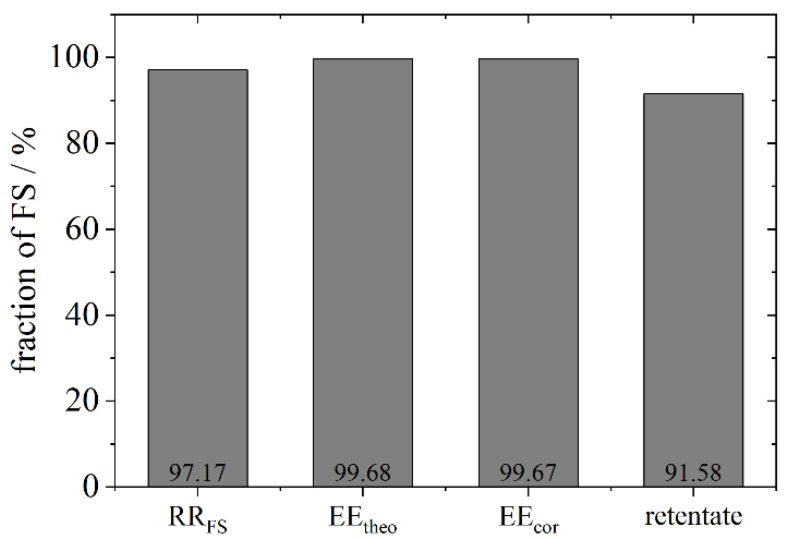
Depiction of different fractions shown for FS for the phospholipid DPPC with a concentration in the stock suspension of 150 mM. Depicted are the recovery rate RR_FS_ calculated according to Equation (2), the encapsulation efficiency EE_theo_ calculated according to Equation (1), the encapsulation efficiency EE_cor_ calculated according to Equation (3) and the retentate (calculated as in Equation (3)) after treatment of the Vivaspin^®^ membrane with perchloric acid.

A separation of free and encapsulated FS is indispensable and was carried out in an additional experimental set up with a Vivaspin 500^®^, exemplarily for the phospholipid DPPC with a concentration of 150 mM in ddH_2_O. The absorption spectrum of the filtrate containing the free FS compares to the spectrum of the diluted stock solution of FS and allows the determination of the encapsulation efficiency. In addition, the retentate was treated with perchloric acid to solubilise the liposomes from the Vivaspin^®^ membrane. For best results, the membrane was washed twice with the same solution. The encapsulation efficiency calculated with different methods is shown in Figure 5. The RR_FS_ is almost 100% in comparison to the initial concentration (97%). As the filtrate only contains small amounts of FS and the RR_FS_ is high, both the EE_theo_ and EE_cor_ equal 99%. The retentate left on the membrane of the Vivaspin^®^ measures 92% (calculated as in Equation (3)) which differs by 8% from the EE calculated from the filtrate. Thus, in the following depictions, results are based on the filtrate. 

In the following, the encapsulation efficiency of liposomes produced from different phospholipid concentrations and with the addition of cholesterol were examined (Table 3). Different amounts of DPPC in the stock solution are able to encapsulate different amounts of FS: a high concentration of 300 mM leads to an encapsulation efficiency of 84%, calculated from Equation (3), whereas only 50 mM of PL in the stock solution encapsulate a maximum amount of 48%. This leads to the conclusion that higher concentrations of PL used for the production of liposomes encapsulate a greater amount of the model API because of a more stable vesicle. In addition, the losses detected with the phosphorus assay explain that, for a stabilization of water droplets, a greater amount of phospholipids is necessary.

The use of a phospholipid with a shorter chain length (DMPC) shows similar results: a shorter chain length leads to an EE of 96%. However, the RR_FS_ is much lower (by 40%) in comparison to DPPC (cf. Table 2) indicating a bigger loss during the process. 

In general, the addition of cholesterol or negatively charged phospholipid head groups stabilize membranes and liposomes. Hence, a better encapsulation efficiency can be hypothesized. This hypothesis was tested by encapsulating FS with pure DPPG as well as a mixture of DPPC and cholesterol (60:40 mol-%) in comparison to pure DPPC. The filtrate was measured with the UV-Vis. The absorption of the filtrate with DPPG- and DPPC+chol stabilized liposomes is much higher than the measured absorption of the filtrate from DPPC liposomes. Thus, the encapsulation efficiency differs accordingly from 83% for pure DPPC liposomes to 74% when cholesterol is added. Ahmad et al. [21] achieve an encapsulation efficiency of 25.86% for a mixture of PC and chol of 0.9:1, which is increased to 42.34% with a higher PC proportion of 1.1:1. A decrease of the encapsulation efficiency with an increase of the cholesterol amount was also described by Briguila et al. [22]. As the transition temperature is not elevated, but cholesterol increases the fluidity of the membrane even below the transition temperature, this leads to a higher bending modulus (or bilayer toughness) which is likely to be the explanation for the lower encapsulation efficiency [23]. Cholesterol changes the characteristics of the membrane and thus, is likely to slow down the dynamics of membrane fusion. Chernomordik et al. describe membrane fusion as a function of the membrane characteristics such as the density of fusion proteins and the lipid composition [24]. Two membranes come into contact and form a hemi-fusion before emerging together. The liposome formation is the opposite process: an emulsion droplet contacts the interface and the bilayer is formed while passing the interface. At the moment of detachment of the newly formed liposome from the interface, they are not yet fully closed leading to a leakage of the model API. The rapidity of closure after the detachment defines the encapsulation efficiency. As cholesterol increases the bending modulus, as also described by Pan et al. and Needham et al., membranes need more time to close after detachment from the interface and, thus, lose more of the model API [25,26]. 

Likewise, liposomes produced by the anionic phospholipid DPPG show a smaller encapsulation efficiency of 63% in comparison to liposomes formed by emulsion droplets stabilized with the zwitterionic DPPC. Jing et al. [27] observe a reduction of the lipid bilayer thickness and density due to higher electron net charge density. This phenomenon can be attributed to the negatively charged headgroups of DPPG whose repelling force is stronger than that of the zwitterionic DPPC molecules. Thus, the distance between headgroups and the inclination angle increases. Additionally, the membrane rigidity increases, as described by Faizi et al. [28]. It is assumed that the bilayer is more permeable and FS is released more easily. These results are in accordance with the RR_FS_ for DPPG which also showed more losses.

Zeta potential measurements show a small negative potential for the phospholipids DPPC and DPPC + chol. and a larger negative potential for the negatively charged phospholipid DPPG. We assume that the bending modulus has a stronger influence on the encapsulation than the zeta potential. Nonetheless, for future leakage studies, zeta potential measurements should be taken into account in more detail for different liposome formulations and encapsulated APIs.

### 3.5. Encapsulation Efficiency of BSA and Fluorescently Labelled Dextran under Different Osmotic Conditions

Besides the inquiry regarding the encapsulation of a low molecular weight dye, the question arises as to whether high molecular weight proteins such as BSA show similar results. In addition, future application makes it necessary to investigate the influence of different osmotic conditions that may be present in the blood outside the liposome. Two different set-ups were applied: the phosphate concentration inside the liposome was kept constant at 83 mM while the outer aqueous phase changed from 83 mM to 250 mM; and in a second experiment, the influx and efflux were examined. The influx describes an osmotic situation where a lower salt concentration outside, in comparison to the inside of the liposome, leads to a motion of water ions into the vesicle through the membrane. An efflux is the opposite effect: due to a higher osmotic pressure inside the liposome, the ions move from the inside to the outside. Both cases were performed by creating a maximum difference in salt concentrations (0 mM and 250 mM); additionally, a comparison was implemented with the same conditions inside and outside the liposome.

**Figure 6:**
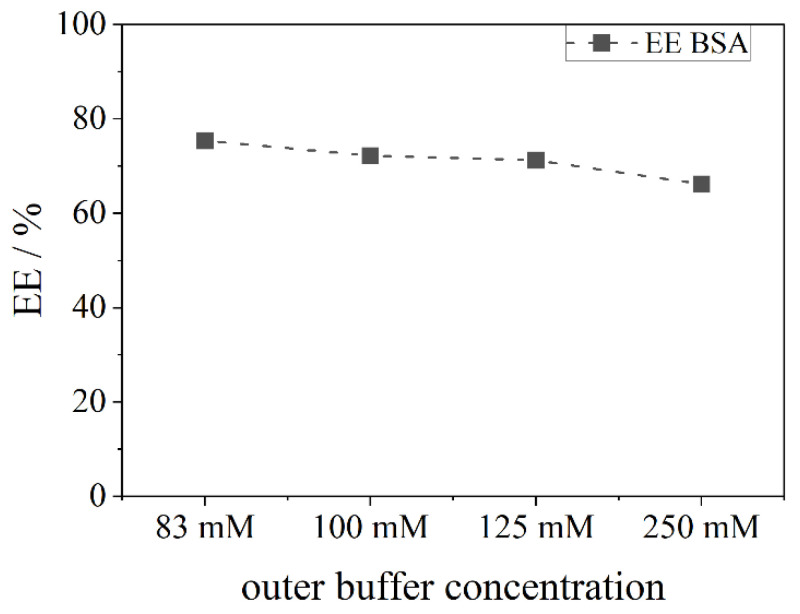
Encapsulation efficiency (EE) of BSA in liposomes prepared from a 150 mM DPPC stock suspension. The inner buffer concentration remains at 83 mM of phosphate buffer, while the outer buffer concentration (the upper aqueous phase) changes from 83 mM to 250 mM.

It is expected that the higher the outside salt concentration, the bigger the efflux and, thus, leaking of the model API. These assumptions are reflected by the results shown in Figure 6. In comparison to FS, the EE of BSA is slightly lower. As the molecular weight is much higher, an encapsulation appears more difficult and thus, a lower EE was expected. However, with the same osmotic conditions inside and outside the liposome (83 mM PB), an EE of 78% can be achieved, which is still higher than efficiencies reported in the literature. The higher the salt concentration outside the liposome and thus, the efflux, the lower the EE. For a maximum difference of salt ions between the inside and outside, an EE of 66% is detected. 

**Figure 7:**
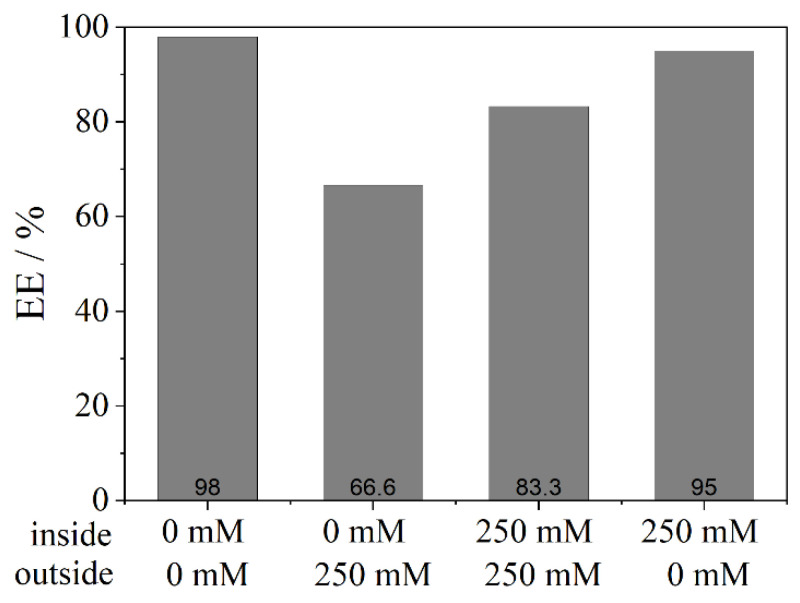
Encapsulation efficiency (EE) of FITC-D mimicking efflux and influx: The first line on the x-axis shows the concentration of phosphate buffer at the inside of the liposome, while the second line equals the concentration of phosphate buffer at the outside aqueous phase after centrifugation. Liposomes were prepared from a 150 mM DPPC stock suspension.

Furthermore, FITC-D was investigated regarding the EE (Figure 7). A similar result is also observed here. While no salt (0 mM) achieves the highest EE (98%), an efflux reduces it by 30%. In comparison, high salt concentrations (250 mM) encapsulate less dextran, but an influx stabilizes the liposome and results in a better EE (95%). These results indicate that, in general, ultrapure water inside and outside the liposome leads to less interference of phospholipids and more stability whereas an efflux destabilizes the lipid vesicles. An influx increases the stability in comparison to the efflux which was not expected, as a strong influx could lead to bursting of liposomes and, thus, set free more of the API.

## 4. Conclusions

The encapsulation not only of low molecular weight substances (FS), but also of macromolecules (BSA and dextrane) with > 60 kDa and without a high affinity to the bilayer, which could enhance the encapsulation, was successfully achieved. The encapsulation efficiency was determined and found to be between 48–98% for the fluorocarbon C_14_F_24_ depending on the used phospholipid. Other fluorocarbons showed a lower EE (20–60%) which revealed that not all fluorocarbons encapsulate the model APIs equally well. A higher density of the fluorocarbon is to be favoured for the process. These achievements were possible due to the novel preparation method of liposomes by using a centrifuge to transfer droplets from a w/fc nano-emulsion. The results show that compounds which stabilize a membrane, such as cholesterol or charged phospholipids, have a negative effect on the encapsulation efficiency. This effect is likely to be due to the greater bending elasticity of the membrane which leads to a slower closure of the vesicle after the detachment of the liposome from the interface. Hence, the leakage increases. For further stabilization of liposomes and for mimicking a future application in the blood, osmotic conditions were imitated. An efflux negatively affects the encapsulation efficiency, an influx leads to a better encapsulation than under efflux conditions, while best results were found for pure water inside and outside.

Thus, the results and the liposome preparation method show not only the capacity for large-scale application, but the method of analysis is also applicable for a scale-up. These data demonstrate a promising alternative for industry in producing liposomes for pharmaceutical application, which additionally hold the capacity to encapsulate high amounts of macromolecules.

## Figures and Tables

**Table 1:** Concentration of phospholipids found in the initial stock suspension (A), the measured phospholipid amount in % based on the initial amount (B), the amount found in the re-dispersed upper aqueous phase after centrifugation (C) and at the wall of the reaction tube after washing with the same volume of perchloric acid as the previous emulsion (D). (E) displays the sum of (C) and (D) while (F) is the total amount of phospholipids found after centrifugation based on (A) and equals the RR_PL_ according to Equation (2).

Phospholipid	(A)/mM	(B)/%	(C)/mM	(D)/mM	(E)/mM	(F)/%
150 mM DPPC	108.72	72.48	0.65	0.17	0.82	75.58
300 mM DPPC	215.58	71.86	1.11	0.33	1.44	66.72

**Table 2:** Total amount of FS found after centrifugation and determined by applying perchloric acid to the aqueous phase. The recovery rate RR_FS_ is calculated from Equation (2) for different phospholipids and concentrations.

Concentration, Phospholipid	RR_FS_/%
50 mM DPPC	48.13
150 mM DPPC	91.97
300 mM DPPC	91.46
150 mM DMPC	45.08
150 mM DPPG	81.26
300 mM DPPG	81.51

**Table 3:** Encapsulation efficiency EE_cor_ according to Equation (3) of different phospholipids and concentrations and the zeta potential ζ in mV.

Concentration, Phospholipid	EE_cor_/%	ζ Potential/mV
50 mM DPPC	48.1	
150 mM DPPC	80.4	−2.4 ± 0.5
300 mM DPPC	83.6	
300 mM DPPC + Cholesterol (60:40)	73.7	−3.4 ± 0.6
150 mM DMPC	96.8	
300 mM DPPG	63.1	−44.4 ± 1.0

## Data Availability

Not applicable.
